# Editorial: Therapeutic potential of natural products-based drugs in regulation of lipid metabolism

**DOI:** 10.3389/fphar.2023.1216367

**Published:** 2023-06-07

**Authors:** Aleksandar Rašković, Ana Tomas, Nebojša Stilinović, Nataša Duborija Kovačević, Hani Al-Salami

**Affiliations:** ^1^ Department of Pharmacology and Toxicology, Faculty of Medicine, University of Novi Sad, Novi Sad, Serbia; ^2^ Department of Pharmacology and Clinical Pharmacology, Faculty of Medicine, University of Montenegro, Podgorica, Montenegro; ^3^ The Biotechnology and Drug Development Research Laboratory, Curtin Medical School and Curtin Health Innovation Research Institute, Curtin University, Bentley, WA, Australia

**Keywords:** plant products, atherosclerosis, dyslipidemia, ethnopharmacology, pharmacologic (drug) therapy

Disturbances in lipid metabolism have been implicated in many chronic conditions. Alterations in cholesterol homeostasis leading to changes in plasma lipid profiles which are an important factor contributing to increasing morbidity of metabolic syndrome, atherosclerosis, diabetes mellitus, and non-alcoholic fatty liver disease (Rašković et al, 2019; Shaito et al, 2020). Dyslipidemias also play a critical role in vascular and neurological complications leading to cerebrovascular disease. Many studies identified high potential of herbal and traditional medicine and other natural products-based drugs in prevention and treatment of metabolic disorders through modulation of lipid homeostasis. Recent years brought on a considerable progress in elucidating the mechanism behind dyslipidemia. A number of studies showed that dyslipidemia and gut microbiota changes are closely intertwined. That has opened avenues for its management by targeting the gut microbiota (Lei et al, 2022) and need to include the assessment of effects of examined products on microbiota composition. Still, plant kingdom remains an under-explored area in terms of finding the exact species useful in preventing and treating disorders associated with changes in lipid metabolism and dissecting exact cellular and molecular beneficial mechanisms.

Considering this fact, the current Research Topic was dedicated to studies of xenobiotics of plant origin with beneficial effects on lipid metabolism and dyslipidemia. An interesting and eclectic collection of articles in this Research Topic includes several pre-clinical studies dedicated to underpinning exact mechanisms behind beneficial effects of products with history of use in traditional medicine, identifying important mechanisms responsible for the observed effects, including influence of herbal extracts on the interplay between gut microbiota and lipid metabolism. Together with a comprehensive overview of traditional Chinese medicine products and components used for dyslipidemias already on the market, as well as one opinion article, all revolving around role of materials of plant origin in lipid metabolism, these papers point to potential and space for their further research in dyslipidemias.

In article by Ji et al., the mechanism of hypolipidemic effects of Fermented *Rosa roxburghii* Tratt juice (FRRT) was explored using 16S rDNA sequencing for microbiota composition and metabolomic profiling of bile acid, amino acid and lipids in high-fat diet induced hyperlipidemia in rats. FRRT treatment was associated with lower body weight in animals, and changes in lipid profiles, with reduction of total cholesterol, triacylglycerol and low-density lipoprotein cholesterol levels, and increase in high-density lipoprotein cholesterol. Results suggest that the serum lipid changes induced by FRRT are in correlation with the alterations in gut microbiota (i.e., *Prevotella, Oscillospira,* Paraprevotellaceae*_Prevotella, Ruminococcus*) and its associated metabolites (amino acid metabolites, i.e., glutamine, glutamic acid, alanine, *etc.*, fatty acid metabolites, i.e., dodecanoic acid, myristic acid, decanoic acid, *etc.*, and bile acids metabolites such as HDCA, LCA, DCA, *etc.*). After FRRT treatment, an increase of several healthy microbial genera and the decrease of potentially pathogenic microorganisms were observed.

A second study exploring the interplay between gut microbiota and lipid levels in response to treatment with aqueous extract of *Asparagus cochinchinensis* root (ACE) in high-fat diet-induced obesity in mice (Luo et al.). ACE administration significantly decreased the weight gain and relieved dyslipidemia, improved glucose tolerance and insulin resistance, remarkably decreased inflammation and lipogenesis in the liver and adipose tissue. In addition, ACE induced changes in gut microbiota with decreased *Proteobacteria*, reduced abundance of *Clostridiaceae* with no effect on *Firmicutes/Bacteroidetes* ratio. These findings show that traditional medicine products are able to affect gut microbiota, significantly modifying the structure and composition of gut bacteria, which is altered in the high-fat diet-induced metabolic disorders.

A study by Ling-Fei et al. explored traditional Uyghur medicine (*Hyssopus cuspidatus* Boriss extract (SXCF)), focusing on the effect of metabolism of lipid mediators (LMs), and combining inflammatory factors such as TNF-α, IL-4, IL-10, IFN-γ, serological indicators, behavioral changes, and pathological findings in ovalbumin-induced asthma model in mice. SXCF treatment was associated with dose-dependent partial restoration of the perturbed LMs metabolic network. The authors identified TXB2, 5-HETE, and HODEs as LMs crucial for the effects of SXCF. SXCF treatment significantly reversed the abnormal upregulation of LMs, which results in reversal of changes in cytokine levels (i.e., TNF-α and IL-4) and reduced infiltration of lung tissue with inflammatory cells, finally leading to the reduction of asthma symptoms.

Many traditional products have been on the market for a long time. They have well established ethnopharmacological therapeutic potential and are widely used. The exact identification of those employed in the context of dyslipidemias was provided in a study by Chi et al. The study reports on an overview of the 91 hypolipidemic formulas sold by 116 traditional Chinese medicine pharmacies in Taiwan. These products were analyzed and the core drug combination of the hypolipidemic formulas were identified. This comprehensive study also explored the strategy influencing selection of commonly used medicinal materials in these products. More than 80 traditional Chinese medicinal materials were identified, belonging to 43 families, predominantly Lamiaceae. Specifically, core materials were *Astragalus mongholicus* Bunge and *Crataegus pinnatifida* Bunge, and the core formulae were Bu-Yang-Huan-Wu-Tang and Xie-Fu-Zhu-Yu-Tang. Despite long history of traditional use of these products, hypolipidemic mechanisms remain unclear and this study provides an insight into most important formulae, offering inspiration for further studies.

Despite the vast volumes of research into xenobiotics of herbal origin in the context of lipid metabolism disturbances, many attempts to move from bench to the bedside were not completely successful. An opinion article by Nemet et al. offered the author standpoint of importance, efficacy and feasibility and need for further studies on products based on carob for the treatment of dyslipidemia. By summarizing evidence from pre-clinical and clinical studies authors infer the mechanism behind hypolipidemic effects of carob pulp to be the synergistic action of its two key components: insoluble fiber and polyphenols. These compounds affect gastrointestinal tract, liver, adipose tissue and exert antioxidative, anti-inflammatory, and vascular-protective activity. Authors are of the opinion that carob is a promising product, but its clinical use is far from established. Carob supplementation could be considered as an addition to healthy lifestyle changes in mild cases of dyslipidemia or as an adjunct to standard hypolipidemic therapy. However, considering substantial evidence for lipid-modifying effects of carob, authors call for more detailed studies isolated active principles from carob which reveal potential target molecules thereby potentially accelerating drug discovery.

Overall, the articles included in this Research Topic provided the current status, challenges, and prospects of research on natural products in lipid metabolism and cholesterol homeostasis.
